# Exploring the costs and outcomes of sexually transmitted infection (STI) screening interventions targeting men in football club settings: preliminary cost-consequence analysis of the *SPORTSMART* pilot randomised controlled trial

**DOI:** 10.1136/sextrans-2014-051715

**Published:** 2014-12-15

**Authors:** Louise J Jackson, Tracy E Roberts, Sebastian S Fuller, Lorna J Sutcliffe, John M Saunders, Andrew J Copas, Catherine H Mercer, Jackie A Cassell, Claudia S Estcourt

**Affiliations:** 1 Health Economics Unit, School of Population and Health Sciences, College of Medical and Dental Sciences, University of Birmingham, Birmingham, UK; 2 Centre for Immunology & Infectious Disease: Sexual Health & HIV, Blizard Institute, Queen Mary, University of London, Barts and the London School of Medicine and Dentistry, London, UK; 3 Centre for Sexual Health and HIV Research, Faculty of Population Health Sciences, University College London, London, UK; 4 Division of Primary Care & Public Health, Brighton and Sussex Medical School, University of Brighton, Falmer, Brighton, UK

**Keywords:** ECONOMIC ANALYSIS, SCREENING, OUTREACH SERVICES, CHLAMYDIA INFECTION

## Abstract

**Background:**

The objective of this study was to compare the costs and outcomes of two sexually transmitted infection (STI) screening interventions targeted at men in football club settings in England, including screening promoted by team captains.

**Methods:**

A comparison of costs and outcomes was undertaken alongside a pilot cluster randomised control trial involving three trial arms: (1) captain-led and poster STI screening promotion; (2) sexual health advisor-led and poster STI screening promotion and (3) poster-only STI screening promotion (control/comparator). For all study arms, resource use and cost data were collected prospectively.

**Results:**

There was considerable variation in uptake rates between clubs, but results were broadly comparable across study arms with 50% of men accepting the screening offer in the captain-led arm, 67% in the sexual health advisor-led arm and 61% in the poster-only control arm. The overall costs associated with the intervention arms were similar. The average cost per player tested was comparable, with the average cost per player tested for the captain-led promotion estimated to be £88.99 compared with £88.33 for the sexual health advisor-led promotion and £81.87 for the poster-only (control) arm.

**Conclusions:**

Costs and outcomes were similar across intervention arms. The target sample size was not achieved, and we found a greater than anticipated variability between clubs in the acceptability of screening, which limited our ability to estimate acceptability for intervention arms. Further evidence is needed about the public health benefits associated with screening interventions in non-clinical settings so that their cost-effectiveness can be fully evaluated.

## Introduction

Young people have the highest risk for sexually transmitted infections (STIs) in the UK, and attempts to engage young men in effective screening have proven to be particularly challenging. Public Health England estimated that 16% of young men aged 15–24 years were screened for chlamydia during 2012 compared with 35% of young women (assuming one test per person).^1^ A range of strategies have been proposed to increase screening participation among men, including outreach in non-clinical settings such as sports venues.^2^ Recent research has suggested that people without a healthcare background can successfully promote certain health behaviours among their peers.^3^ Among young men in England, football has the highest levels of participation for a team sport.^4^ The SPORTSMART pilot trial was designed to develop and evaluate the feasibility and acceptability of two replicable models for promoting STI screening in football clubs (specifically, *Chlamydia trachomatis* and *Neisseria gonorrhoeae* screening), including screening promoted by team captains.

The success of any new intervention in increasing screening uptake needs to be balanced against the resources required to achieve the desired outcome, and additional costs must be evaluated in terms of any additional benefits that can be attributed to them. The objectives of this economic analysis were to obtain cost and outcome data for the alternative screening interventions developed by the SPORTSMART pilot trial and to use these data in a preliminary economic evaluation.

## Methods

### Pilot trial

The methods and results of the pilot trial are reported in detail elsewhere.^5^ In brief, a cluster randomised control trial (RCT) design was used involving the allocation of clubs, each with two teams, to one of three trial arms: (1) captain-led and poster STI screening promotion arm; (2) sexual health advisor-led and poster STI screening promotion arm and (3) poster-only STI screening promotion control/comparator arm. The participants were men aged 18 years and over within six amateur football clubs in London. Eligible football clubs were grouped by similar characteristics into three pairs, and then each of the pairs was randomised to a study arm.

The interventions were delivered during the prematch team briefing. For the captain-led promotion, the captain delivered a one-time standardised promotion talk of <5 min duration, handed each player a specially designed football-themed sample collection kit and answered any questions from players. In the sexual health advisor-led arm, the standardised promotion talk was delivered by a sexual health advisor from the study clinic. In the poster-only arm, specially designed posters were displayed and kits were made readily available, but no verbal information was given. If men chose to participate, they completed a sample collection kit and placed the completed kit in a secure collection box at the club within an hour after the match ended or they had the option of participating at a later date and posting their sample back to clinic in a discrete postage-paid envelope. Provision of test results and appropriate clinical follow-up was undertaken by the clinical team in the study clinic (off-site), according to routine clinical practice. This included notification of provision of test results via a text message (SMS) by clinic staff.

The primary outcome was the proportion of eligible men accepting the screening offer. The target was to achieve a sample size of 200 men as this would allow the overall acceptance rate to be estimated within 7% if the rate was 50% (ie, a 95% CI 43% to 57%) and within 5% if the rate was higher or lower, assuming minimal variability between clubs.

### Economic analysis: overview

For all arms of the trial, resource use data were collected prospectively and unit costs were applied. There were several elements that were common to all of the intervention arms; these related to the recruitment and briefing of the clubs, the materials used within the interventions, and the collection and processing of completed samples. The time taken to recruit, brief and prepare the clubs for the screening intervention was recorded and staffing costs estimated using the Unit Costs of Health and Social Care 2013.^6^ Costs associated with travel to the clubs to prepare for the intervention were also included.

Specially designed posters, football-themed sample kit packs and themed collection containers were used at all locations to promote screening and provide the equipment necessary for participation. We assumed that all these elements were an essential part of the intervention and included their costs in our estimates. We assumed that designs and logos could be reused over a period of 3 years until they became outdated, and thus annuitised all design and editing costs for 3 years at an interest rate of 3%.^7^ We assumed an even number of test kits across intervention arms as data recorded in the trial demonstrated that similar quantities of testing materials were used across arms, as unused items were reused in other clubs. Transport costs associated with returning the completed player samples to the study clinic (off-site) were also included. Although players were given the option to return their samples by post, only one sample was returned by this method, and for the base case, we assumed that the secure collection box would be the only method of specimen return provided.

Additional facilities were required for storage of the samples before they were sent to the laboratory for processing. We assumed that such additional storage would be needed if the intervention were rolled out and that these facilities could be reused over a period of 3 years; we included annuitised costs accordingly. Costs associated with processing samples were estimated using the cross charge between the processing laboratory and the study clinic. Staff time associated with patient administration at the clinic was recorded and costs estimated. We also included costs associated with notifying players about their results and any further costs associated with patient consultation and treatment. For all trial arms, data were collected on direct health service costs and some of the private costs incurred by the players and captains. The main analysis was conducted from the perspective of the health service (National Health Service).

### Resource use and cost definition

#### Captain- and poster-promoted screening

The estimated cost of the captain-led screening intervention included costs for a member of staff (a healthcare assistant) from the clinic undertaking the sample processing and notification to be on site before and after the intervention to deliver and prepare all the materials and to facilitate the safe return of the completed samples to the clinic, based on recorded practice within the pilot trial and clinical governance requirements. For the base case, it was assumed that the time taken by the team captain to prepare for and deliver the intervention was forgone leisure time and would not impact on health service costs. However, the effect of including these costs or some kind of financial incentive for the captain was analysed as part of the sensitivity analysis.

#### Sexual health advisor- and poster-promoted screening

The estimated costs for the health advisor-promoted intervention included costs for a sexual health advisor to lead the screening promotion. We assumed that the health advisor would also take the materials to the club, prepare the promotion and ensure the safe return of completed specimen samples to the clinic, in accordance with trial processes and clinical governance requirements and hence included time and travel costs in our estimates.

#### Poster-promoted screening only (control)

As for the captain-led arm, we assumed that a member of staff (a healthcare assistant) from the clinic undertaking the testing and notification would need to be on site before and after the promotion and included costs accordingly.

### Analysis

We conducted a cost-consequences analysis that involves comparing the costs and outcomes associated with all three interventions separately.^8^ This kind of analysis is more appropriate than a full economic evaluation because the current study is a pilot only and a full RCT has not been carried out.

We assessed costs and outcomes in a disaggregated manner for each intervention arm to establish whether any showed clear dominance. Dominance is judged to have occurred when one intervention costs less but is more effective, in terms of the outcome achieved, compared with a different intervention. Conversely, an intervention is dominated if it costs more but is less effective than the comparator. We examined costs and consequences for all three arms. The main analysis is based on the outcome of whether the player accepted the offer of screening. All cost data reported are presented in British pounds in 2012/2013 prices.

A series of one-way deterministic sensitivity analyses were carried out. Uncertainties around all key cost and outcome parameters were analysed, and plausible ranges were specified using information from the trial and from the literature. These analyses included (a) reducing club recruitment time substantially to 4 h per club by developing a higher-level agreement with the Football Association; (b) including an incentive of £1000 for each club to help maximise participation (to reflect practice within the study); (c) including broader societal costs associated with captain participation in screening, with the assumption that the time taken to participate in the intervention was forgone leisure time, valued at 40% of the median hourly wage;^9^ ^10^ (d) reducing intervention costs for the poster-only control arm to analyse the impacts of different staffing arrangements; (e) adjusting the cost of the test kit boxes to account for the logo and design costs associated with unused boxes; (f) increasing sample processing costs and (g) varying uptake levels by study arm. Further sensitivity analyses were carried out but are not reported.

## Results

Across all three of the trial arms, 153 men received the intervention and 90 of them accepted the screening offer (59%, 95% CI 35% to 79%, using a robust SE to acknowledge the clustering of participants by club). There was considerable variation in the uptake rates between individual clubs, but results were broadly comparable across study arms (table 1). For the captain-led arm, 56 men received the promotion and 28 accepted (50%). For the health advisor-led arm, 46 men attended the promotion and 31 accepted (67%). For the poster-only control arm, 51 men received the promotion and 31 accepted (61%). There were no positive test results for chlamydia or gonorrhoea in any of the study arms.

**Table 1:** Screening uptake for clubs within the study arms

Study arm	Club	Players in changing room	Completed kits returned	Percentage of return
Captain-led	A	26	10	38.5
Captain-led	B	30	18	60.0
		*56*	*28*	*50.0*
Health advisor-led	A	24	10	41.7
Health advisor-led	B	22	21	95.5
		*46*	*31*	*67.4*
Poster-only (control)	A	24	20	83.3
Poster-only (control)	B	27	11	40.7
		*51*	*31*	*60.8*
Total	All	153	90	58.8

Full costs for each of the intervention arms are shown in table 2, and further details are included in online supplementary appendix 1. The results of the pilot trial suggested that total costs were similar across all of the intervention arms, with the total costs of the captain-promoted screening intervention estimated to be £2491.61 compared with £2738.09 for the health advisor-led arm and £2538.09 for the poster-only arm. Overall costs were similar because the highest proportion of costs related to fixed costs, such as staff time for recruiting and briefing the clubs and the equipment required for delivering the promotion.

**Table 2:** Health service costs for intervention arms (two clubs per arm)

Resources used	Cost item	Unit cost £*	N	Total cost £*
Intervention costs				
Recruitment of club	Per club	516.88	2	1033.75
Poster pack†	Per pack	53.92	2	107.85
Test kit†	Per player	5.66	46	260.36
Promotion	Per club	Captain-led: 125.00	2	Captain-led: 250.00
		Health advisor-led: 225.00		Health advisor-led: 450.00
		Poster-only: 125.00		Poster-only: 250.00
Specimen collection box†	Per club	55.62	2	111.25
Transport of specimen collection box	Per club	135.64	2	271.28
Processing costs				
Additional storage facilities‡	Per club	11.63	2	23.26
Sample processing	Per player tested	10.79	Captain-led: 28	302.12
			Health advisor-led: 31	334.49
			Poster-only: 31	334.49
Patient admin and notification of results	Per player tested	4.71	Captain-led: 28	131.74
			Health advisor-led: 31	145.86
			Poster-only: 31	145.86
Total cost				Captain-led: 2491.61
				Health advisor-led: 2738.09
				Poster-only: 2538.09

*Costs are UK£ (2012/2013).

†Includes costs for the first year of the design elements of the posters, test kit box, pens and specimen collection boxes, annuitised at 3% for 3 years.

‡Includes costs for the first year of the storage facilities, annuitised at 3% for 3 years.

For all three intervention arms, costs were compared with the main outcome of screening uptake (table 3). The average cost per player tested was comparable across the trial arms using the base case results, with the average cost per player tested for the captain-led promotion estimated to be £88.99 compared with £88.33 for the sexual health advisor-led screening promotion and £81.87 for the poster-only (control) arm.

**Table 3:** Comparison of costs and outcomes for the intervention arms

Intervention arm*	Total cost £†	Number of players tested	Per cent accepting screening offer	Average cost per player screened £†
Captain-led	2491.61	28	50	88.99
Health advisor-led	2738.09	31	67	88.33
Poster-only (control)	2538.09	31	61	81.87

*Includes costs and outcomes for both clubs in each trial arm.†Costs are UK£ (2012/2013).

### Sensitivity analysis

As demonstrated in table 4, the results were as follows: (a) decreasing the time needed for club recruitment reduced overall costs, with the cost per player screened ranging from £60.14 to £66.59; (b) including an incentive in our analysis increased overall costs for all trial arms; (c) including costs for team captains to deliver the promotion made the captain-led arm slightly more expensive, however, as process evaluation revealed that team captains had also informally promoted the screening intervention in other trial arms including these costs for the captain-led arm alone may not be justified; (d) reducing intervention costs for the poster control arm led to a reduction in overall costs for this arm; (e) increasing the costs associated with the test kit boxes (to adjust for costs associated with unused boxes) increased total costs for all intervention arms with estimates per player screened ranging from £84.26 to £91.63; (f) increasing sample processing costs increased costs for all trial arms and (g) varying uptake levels had an effect on the result, emphasising the importance of an accurate estimate of effectiveness.

**Table 4:** Sensitivity analysis: selected results

	Original value	Revised value	Captain-led arm: total cost* (average cost per player screened)	Health advisor-led arm: total cost* (average cost per player screened)	Poster-only arm: total cost* (average cost per player screened)
Base case			£2491.61 (£88.99)	£2738.09 (£88.33)	£2538.09 (£81.87)
(a) Reducing club recruitment time to 4 h per club	£516.88†	£180†	£1817.86 (£64.92)	£2064.34 (£66.59)	£1864.34 (£60.14)
(b) Including £1000 incentives for each club		£1000†	£4491.61 (£160.41)	£4738.09 (£152.84)	£4538.09 (£146.39)
(c) Including costs for team captains to deliver promotion		£2.68†	£2496.87 (£89.17)		
(d) Reducing promotion costs for the poster-only control arm	£125.00†	£50.00†			£2388.09 (£77.04)
(e) Increasing costs for testing boxes	£5.66‡	£7.27‡	£2565.67 (£91.63)	£2812.15 (£90.71)	£2612.15 (£84.26)
(f) Increasing sample processing costs	£10.79‡	£16.19‡	£2642.67 (£94.38)	£2905.34 (£93.72)	£2705.34 (£87.27)
(g) Varying uptake levels by study arm	Captain-led: 50.0% Health advisor- led: 67.4% Poster-only: 60.8%	38.5–95.4%	£2246.10–£2818.77 (£124.78–£64.06)	£2446.10–£3018.77 (£135.89–£68.61)	£2277.09–£2896.24 (£113.85–£59.11)

Costs are UK£ (2012/2013).

*Includes costs and outcomes for both clubs in each trial arm.

†Per club.‡Per item.

## Discussion

This was an exploratory economic evaluation comparing the costs and outcomes of alternative models for promoting screening among young men. The results as a whole suggest that all these methods of screening promotion are acceptable to players within amateur football clubs, with 153 men receiving the intervention and 90 accepting the offer of screening (59%, 95% CI 35% to 79%). The overall costs associated with the intervention arms were similar. The outcome of average cost per player screened was comparable across all arms, with the average cost per player tested for the captain-led promotion estimated to be £88.99 compared with £88.33 for the sexual health advisor-led screening promotion and £81.87 for the poster-only (control) arm. This outcome is affected by the estimate of the number of players accepting screening, and as our ability to estimate uptake for any single intervention arm is limited, drawing conclusions about the relative costs and consequences of the interventions is not justified. No intervention model can be judged to be dominant, and the average cost per player tested can be seen as comparable across all of the study arms.

It might have been expected that the costs associated with the poster-only control and captain-led arms would be lower than for the health advisor-led arm. However, costs were found to be similar due to the need for a member of clinic staff to be on site and ensure the return of samples to clinic, to meet clinical guidelines. In the event of a rollout of the trial, a satisfactory alternative to this arrangement might be found.

This study has several limitations. It was difficult to draw firm conclusions about the relative cost-effectiveness of the interventions in the trial as screening uptake could not be estimated with precision for any single intervention arm. Two clubs were randomised to each intervention arm; however, we found a greater than anticipated variability between clubs in the acceptability of screening, which limited our ability to estimate acceptability for intervention arms. In addition, the overall target sample size was not achieved due to difficulties in recruitment linked to poor weather and rescheduled matches. We did not capture additional downstream testing that may have occurred as a result of the intervention, and so uptake of STI testing might have been underestimated. As the trial did not identify any positive cases of chlamydia or gonorrhoea, it was not possible to estimate a cost per case diagnosed.

Within the economic analysis, some assumptions were made about how the interventions would operate if they were rolled out, and although these were examined within a sensitivity analysis, they would need to be tested at a larger scale. A one-way deterministic sensitivity analysis was carried out since this is a preliminary economic analysis alongside a pilot trial and a full probabilistic sensitivity analysis would not be appropriate because of the small sample size and the heterogeneity of uptake rates at club level. Further uncertainties around cost and outcomes parameters would need to be analysed if a full RCT was conducted. Finally, it was intended that players in the control arm would be *uninfluenced* by team captains, but their enthusiasm for the intervention meant that this was not possible, and captains encouraged players to participate in screening via regular team information emails.

The strength of this study is that detailed data on costs and resource use were collected which can inform similar interventions in this area and enable comparisons with other research findings. In addition, data were collected on the number of players who attended the screening promotion events, and thus it is possible to estimate the level of uptake of screening. Often with such health promotion interventions, the number of people exposed to a particular intervention is unknown.

Very little information exists about the cost-effectiveness of screening programmes in non-clinical settings. A recent systematic review of studies concerned with chlamydia and gonorrhoea screening outreach programmes only identified three with information on costs.^11^ Buhrer-Skinner *et al*^12^ calculated a cost per test carried out for outreach clinics in Australia but did not include staff time, transport and setup costs. Morris *et al* calculated the costs associated with two Californian youth programmes but did not include additional time associated with volunteer input.^13^ Detailed costings were also provided by a study evaluating the cost-effectiveness of a multifaceted community intervention to increase screening in Stockholm.^14^ However, due to the nature of the intervention, which involved a large-scale publicity campaign and expanded access to testing facilities, it is difficult to compare these results with the SPORTSMART study. The findings of a costing study of chlamydia screening within primary care suggest that the costs per case screened for the SPORTSMART study are higher than would usually be expected in a UK primary care setting.^15^ However, the additional public health benefits associated with outreach activities would also need to be taken into account and achieving adequate coverage of screening in primary care, especially in men, is challenging.

### Conclusions

This preliminary economic evaluation has shown that similar costs and outcomes were demonstrated across all three study arms. The fact that the control arm was unintentionally ‘enhanced’ by team captains suggests that they can have an important influence on screening uptake, irrespective of whether they take on a formal role in promoting the intervention and the potential costs associated with their informal and formal input need to be carefully considered. Further research is needed to explore the public health benefits associated with screening interventions in non-clinical settings so that their cost-effectiveness can be fully evaluated. This would allow the potentially higher costs associated with such ‘outreach’ activities to be weighed against their wider health benefits so that policymakers can be fully informed about which approaches offer the best value for money.

**Key messages**

- Although acceptance rates were highly variable between clubs, levels of uptake were broadly comparable across all study arms.
- The overall costs associated with the intervention arms were similar.
- No intervention model was judged to be dominant, and the average cost per player tested was comparable across all of the study arms.
- Further research is needed to investigate the public health benefits associated with screening interventions in non-clinical settings so that their cost-effectiveness can be fully evaluated.

## Supplementary Material

Web supplement
