# Supplementary material for: Exploring the costs and outcomes of sexually transmitted infection (STI) screening interventions targeting men in football club settings: preliminary cost-consequence analysis of the SPORTSMART pilot randomised controlled trial
Source: Sex Transm Infect. 2014 Dec 15;91(2):100–5. doi: 10.1136/sextrans-2014-051715 (PMC4345770; doi:10.1136/sextrans-2014-051715)
Supplement: Web supplement [file sextrans-2014-051715-s1.pdf]

APPENDIX 1 (web only)

Cost details for test kit boxes

Item	Unit cost (including VAT & shipping costs) £*	n	Cost per pack £*
Biohazard envelope	£1.05	1	£1.05
Test kit outer box**	£2.23	1	£2.23
Urine transporters	£0.66	1	£0.66
Urine container (30 ml)	£0.15	1	£0.15
Test request form	£0.03	1	£0.03
Pen**	£0.85	1	£0.85
Condoms	£0.22	3	£0.66
Information sheet	£0.03	1	£0.03
**Total cost of one test kit*** **			**£5.66**

* Costs are £UK (2012/13).
**Includes costs for the first year of the design elements of the test kit box and pens, annuitized at 3% for 3 years. Costs include VAT where applicable.
***Based on costs recorded in the trial. Assumes that unused test kit elements can be utilised in the clinic.
